# Pseudosarcomatous myofibroblastic lesion of the urinary bladder: A rare entity posing a diagnostic challenge and therapeutic dilemma

**DOI:** 10.1186/1746-1596-3-11

**Published:** 2008-03-13

**Authors:** Alexandros Lekas, Aikaterini Parasi, Thomas G Papathomas, Athanasios G Papatsoris, Maria Rozaria Mennonna, Michail Chrisofos, Charalambos Deliveliotis, Andreas C Lazaris

**Affiliations:** 1 2nd Department of Urology, Sismanoglion Hospital, School of Medicine, National and Kapodistrian University of Athens, Athens, Greece; 2 Department of Pathology, General Hospital of Nikea, Piraeus, Greece; 3 1st Department of Pathology, School of Medicine, National and Kapodistrian University of Athens, Athens, Greece

## Abstract

**Background:**

Pseudosarcomatous myofibroblastic lesions of the urinary bladder are relatively rare entities of an uncertain pathogenesis and benign indolent nature.

**Case presentation:**

We present an extremely rare case of an ALK-1-positive pseudosarcomatous myofibroblastic lesion of the urinary bladder, which was initially misinterpreted as a low-grade leiomyosarcoma of myxoid subtype on histologic examination owing to prominent atypia, high mitotic activity, abnormal mitotic figures and infiltration of the bladder wall. Although the histologic features were suggestive of a sarcoma, the correct diagnosis was finally established and radical surgical treatment was subsequently avoided. The patient is currently free of disease without any evidence of tumor recurrence or metastasis at 3 years post-operatively.

**Conclusion:**

The key differentiating point rests in distinguishing the aforementioned mass forming lesion from the myxoid subtype of low-grade leiomyosarcoma in order to avoid unnecessary radical therapy.

## Introduction

Non-epithelial tumors account for 2%–5% of all primary urinary bladder neoplasms, with the most common types being rhabdomyosarcoma in patients under the age of 10 years and leiomyosarcoma in adults. The latter has 3 distinct histologic variants: the classical spindle cell subtype, the myxoid subtype and the less frequent epithelioid subtype [1,2]. The key differentiating point rests in distinguishing the myxoid subtype of low-grade leiomyosarcoma from pseudosarcomatous myofibroblastic proliferation, which is a rare entity of benign indolent nature, in order to avoid unnecessary radical therapy [1]. Herein, we report on an extremely rare case of a pseudosarcomatous myofibroblastic lesion of the urinary bladder that displayed prominent atypia and discuss its pathogenesis, histology, differential diagnosis, biologic behavior and pertinent treatment.

## Case presentation

A 36-year-old, postpartum, white female became increasingly symptomatic with sharp, low abdominal pain with the completion of each urination and gross hematuria. She denied night sweats or fever and any history of urinary tract infection, trauma, instrumentation or other urological problems. An ultrasound study of the kidneys and the urinary bladder revealed a broad-based polypoid mass (2.4 × 2.3 × 2.2 cm), located in the right posterolateral wall of the bladder. The patient subsequently underwent a transurethral resection of the aforementioned lesion.

**Figure 1:**
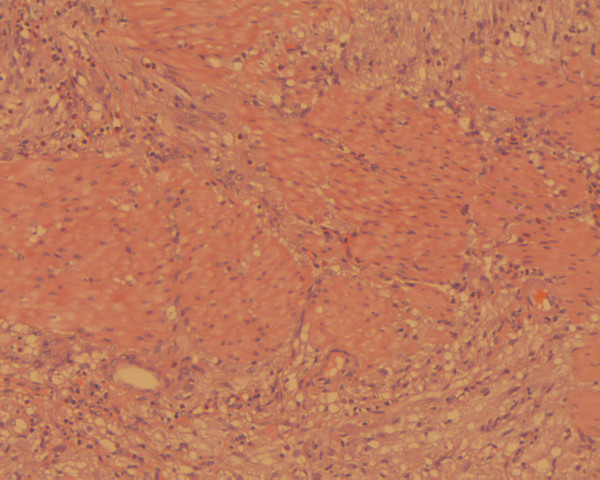
Lesional cells extending between bundles of smooth muscle (hematoxylin-eosin ×200).

**Figure 2:**
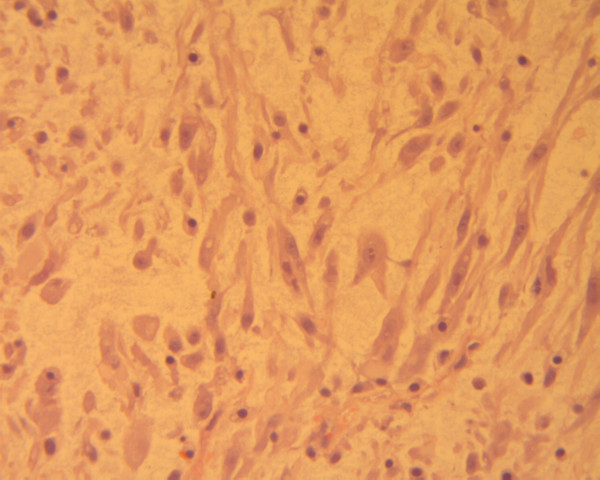
An area of loose spindle-shaped cells, displaying elongated cytoplasmic processes and slight nuclear atypia, in a myxoid background (hematoxylin-eosin ×400).

**Figure 3:**
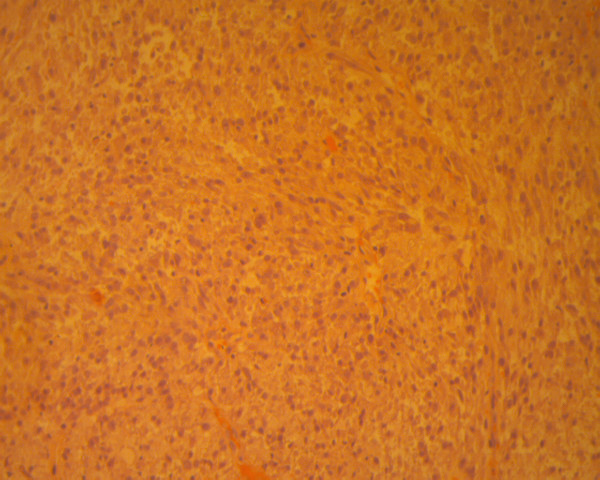
Fascicular arrangement and compact cellularity containing inflammatory infiltrate and conspicuous mitoses (hematoxylin-eosin ×200).

**Figure 4:**
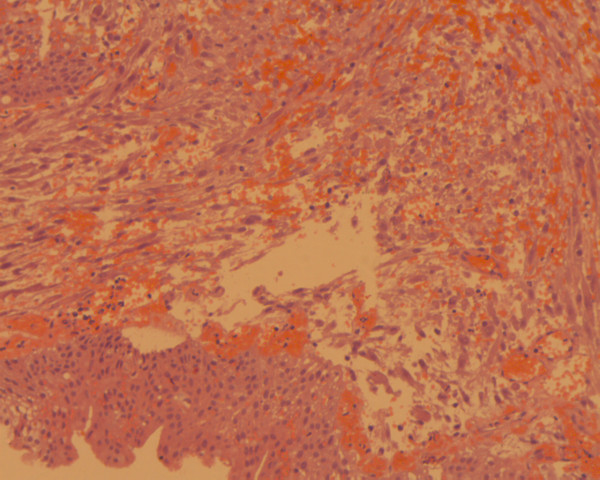
Pseudosarcomatous myofibroblastic tumor exhibiting spindle cells arranged in fascicles with a myxoid stroma and inflammatory cells. Note the intact overlying urothelial mucosa (hematoxylin-eosin ×200).

**Figure 5:**
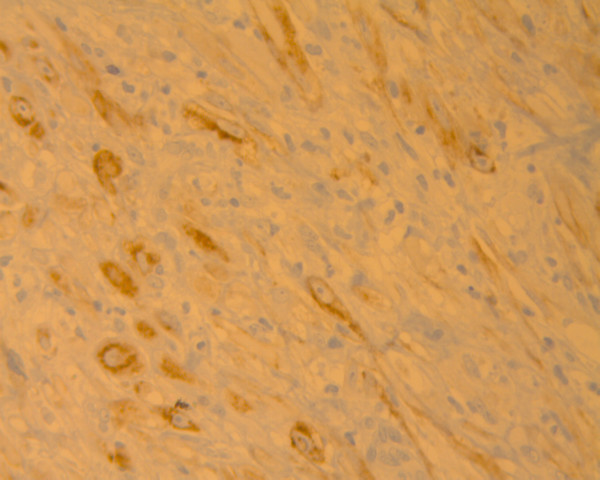
The ALK-1 immunostain demonstrates diffuse cytoplasmic positivity in the lesional cells (immunoperoxidase ×400).

Histologic examination of the excised mass displayed infiltration of the bladder wall (Fig. 1) by an atypical spindle cell proliferation with histologic features of myofibroblasts (Fig. 2) arranged in sheets with focal myxoid areas (Fig. 3), whereas the overlying transitional epithelium was normal (Fig. 4). In particular, the tumor cells demonstrated prominent nucleoli and nuclear pleomorphism; numerous normal mitotic figures (5/10hpf) and a few abnormal ones were seen. By immunohistochemical evaluation, the cells were: (**a**) positive for vimentin and ALK-1 (Fig 5); (**b**) focally positive for cytokeratins 8/18/19, muscle specific and smooth muscle actins, and desmin; (**c**) negative for S-100, CD34, c-Kit (CD117), h-caldesmon and MyoD1.

These histologic findings posed a particular diagnostic problem and yielded a differential diagnosis of pseudosarcomatous myofibroblastic lesion versus low-grade leiomyosarcoma of focally myxoid type. Although the possibility of the former was taken into account, the diagnosis of malignancy could not be avoided, given the nuclear pleomorphism, abnormal mitoses and the infiltrative nature of the lesion. Based on the absence of an *in situ *urothelial neoplasm and the immunophenotype, this was considered to be more likely a smooth muscle tumor than a spindle cell carcinoma. Consequently, further adequate local excision was recommended. At that point, a staging work-up, including a bone scan, abdominal/pelvic MRIs and an intravaginal ultrasound, disclosed no definite evidence of radiographically residual intravesicular tumor and no evidence of extension or metastasis. Due to negative staging work-up and considering the rarity and complexity of the case, the patient was referred for close clinical follow-up and cystoscopic re-evaluation of the bladder excision site for potential residual lesion.

After five months' interval since the initial transurethral resection, a repeat cystoscopy was performed and disclosed scar tissue without any obvious tumor growth; additional biopsies were obtained from the surgical site. Microscopy revealed no sarcoma, but residual foci that simulated a reactive process.

Given that a sarcoma of the bladder would have already shown significant proliferative activity and comparing the initial slides with the re-biopsy slides, we issued a new diagnosis; in particular, we inclined more towards a diagnosis of pseudosarcomatous myofibroblastic lesion instead of a sarcoma. Due to the atypical features encountered and the fact that the biological potential of this entity cannot be accurately assessed, a close follow-up was warranted. The patient did not receive any adjuvant treatment. She is currently free of disease without evidence of tumor recurrence or metastasis by 3-year follow-up.

## Discussion

Non-neoplastic reactive, mesenchymal proliferation of the urinary bladder is a rare, but distinctive entity, which has been referred to as inflammatory pseudotumor (IP) or myofibroblastic tumor (IMT), postoperative spindle cell nodule, nodular fasciitis, pseudosarcomatous myofibroblastic proliferation, pseudomalignant spindle cell proliferation, and pseudosarcomatous or atypical fibromyxoid tumor [3,4]. The unifying feature of these lesions is their tendency to mimic both sarcomas and spindled carcinomas, thus presenting particular diagnostic difficulties in urinary bladder pathology.

The pathogenesis of IMT is still in doubt [4]; some regard this entity as a reactive or inflammatory condition, while others believe that it represents a low-grade mesenchymal malignancy. The latter endorse the low-grade neoplastic nature of this entity owing to (**a**) the occurrence of cases with deep infiltration of the bladder wall and extension into the perivesical soft tissue and (**b**) the demonstration of a non-random chromosomal translocation involving 2p23 that results in the expression of anaplastic lymphoma kinase (ALK) [5]. In addition, the relationship of bladder IMT to lesions called IMT in other anatomic sites remains uncertain [3,6], since bladder lesions are far more likely to express keratin, probably less likely to recur and certainly less likely to metastasize than the latter [3]; albeit, they share similar morphologic features and molecular alterations with these [3,7].

Pseudosarcomatous myofibroblastic lesions of the bladder may occur at any age (range from childhood to elderly patients), but typically occur in adult males [3,8,9]; being in contrast to other reports [10-12] that support a female preponderance. These rare lesions have a non-specific presentation with painless gross hematuria being the most common presenting symptom; other complaints include dysuria, frequency, suprapubic pain, or the discovery of a mass lesion. Their size is quite variable, ranging from a few centimeters up to a reported size of 37.5 cm [9]. Endoscopically and radiographically, these cannot be distinguished from malignant tumors [13]. Cystourethroscopy reveals either an intraluminal (exophytic or polypoid) mass or a submucosal (mural) lesion that may be easily overlooked [10]. When the tumor is sectioned, the mass is relatively gelatinous and soft [11] or has a pale, firm cut surface [10,14] without areas of hemorrhage or necrosis.

IMT contains a mixture of spindle cells showing myofibroblastic differentiation, admixed with variable numbers of inflammatory cells. These display 3 histologic patterns in varying proportions: (a) the myxoid-vascular pattern, characterized by loosely arranged, stellate-to-plump spindle cells in an oedematous, myxoid background with an irregular network of small blood vessels and inflammatory cells, resembling nodular fasciitis or granulation tissue, (b) the compact spindle cell pattern, which consists of a compact interlacing fascicular or storiform spindle cell proliferation with variable myxoid and collagenized regions, accompanied by an inflammatory infiltrate composed largely of plasma cells, resembling fibrous histiocytoma or smooth muscle neoplasms, (c) the hypocellular-fibrous pattern, characterized by platelike collagen, lower cellularity and relatively sparse inflammation with lymphocytes and plasma cells trapped in a dense eosinophilic matrix, resembling a desmoid or scar [15,16]. Microscopically, the lesional spindle cells are bipolar with elongated, eosinophilic cytoplasmic processes, which are devoid of cross-striations, and central oval nuclei characterized by smooth nuclear contours, open chromatin pattern, occasional nucleoli and lack of unequivocal malignant features [10,11]. Anaplastic or pleomorphic features as well as atypical or bizzare mitotic figures are absent; occasional mitoses may be found but the mitotic activity is typically low [11]. Some lesions demonstrate compact cellularity with mitoses, necrosis and bladder wall invasion [3].

Of clinical importance, IMT follows a benign indolent course and conservative management (complete transurethral resection or partial cystectomy) has been reported [1,3,13,17] as treatment of choice. Additional close follow-up (surveillance cystoscopy and biopsy to document resolution) is advised for most cases [3,18] due to the fact that the biological potential of this entity cannot be accurately assessed and its histologic similarity to malignant neoplasms. Moreover, it is critical not to misdiagnose IMT as rhabdomyosarcoma, leiomyosarcoma or sarcomatoid urothelial carcinoma in order to avoid inappropriate radical surgery and/or adjuvant therapy and their attendant complications.

In fact, some leiomyosarcomas of the urinary bladder can express cytokeratin and are partially or extensively myxoid to such a degree that distinction from IMTs may become impossible; albeit, the presence of (**a**) prominent cytological atypia, (**b**) abnormal mitotic figures, (**c**) preferential reactivity for high-molecular-weight caldesmon and the lack of (**a**) a delicate vascular network, (**b**) interspersed inflammatory and red blood cells, and (**c**) ALK-1 immunostaining (usually demonstrated in IMT) may be of particular value in the differential diagnosis [1,3-5,14,19,20]. Similarly, sarcomatoid urothelial carcinomas sometimes show weak or focal immunoreactivity for cytokeratin and display myxoid features leading to potential diagnostic confusion with IMT. Of note, the identification of an in situ or invasive "typical" epithelial component usually allows for their diagnosis; other supportive features in favour of sarcomatoid urothelial carcinomas include prominent cytological atypia, atypical mitoses, non-myxoid areas with marked increased cellularity and usually ALK-1 negativity [3,14]. With regard to embryonal rhabdomyosarcomas in the pediatric setting, these can be distinguished from IMTs by (**a**) exhibiting greater cellularity and increased numbers of atypical mitotic figures, (**b**) the presence of rhabdomyoblasts, a uniform population of small hyperchromatic cells, (**c**) a "cambium layer" (small malignant cells characteristically grouped beneath the epithelium) and (**d**) positive myogenin (Myf4) and MyoD1 immunostainings [5,11,19,20].

Immunohistochemically, it has become apparent that h-caldesmon, myogenin and ALK-1, in contrast to muscle specific and smooth muscle actins, desmin and cytokeratin, can be of great value in the differential diagnosis of vesical IMTs [4,14,20]. As far as ALK-1 cytoplasmic expression is concerned, this has been identified in a range of 8% to 89% of IMT cases in the urinary bladder [20] and in the current report as well; albeit being suggestive of a neoplastic rather than a reactive or inflammatory nature and consistent with abundant mitoses and prominent atypia, the present ALK-positive lesion has neither recurred nor metastasized 3 years post-operatively.

## Competing interests

The author(s) declare that they have no competing interests.

## Authors' contributions

AL participated in the surgical treatment of the patient and drafted the manuscript. AP participated in the pathologic diagnosis. TP drafted the manuscript. AP drafted the manuscript. MRM participated in the pathologic diagnosis. MC participated in the clinical evaluation and follow-up of the patient. CD participated in the clinical evaluation and follow-up of the patient. AL drafted the manuscript.
